# SuperDCA for genome-wide epistasis analysis

**DOI:** 10.1099/mgen.0.000184

**Published:** 2018-05-29

**Authors:** Santeri Puranen, Maiju Pesonen, Johan Pensar, Ying Ying Xu, John A. Lees, Stephen D. Bentley, Nicholas J. Croucher, Jukka Corander

**Affiliations:** ^1^​ Department of Computer Science, Aalto University, FI-00076 Espoo, Finland; ^2^​ Department of Mathematics and Statistics, Helsinki Institute of Information Technology (HIIT), FI-00014 University of Helsinki, Finland; ^3^​ Pathogen Genomics, Wellcome Trust Sanger Institute, Cambridge CB10 1SA, UK; ^4^​ Department of Infectious Disease Epidemiology, Imperial College London, London W2 1PG, UK; ^5^​ Department of Biostatistics, University of Oslo, 0317 Oslo, Norway

**Keywords:** population genomics, epistasis, linkage disequilibrium

## Abstract

The potential for genome-wide modelling of epistasis has recently surfaced given the possibility of sequencing densely sampled populations and the emerging families of statistical interaction models. Direct coupling analysis (DCA) has previously been shown to yield valuable predictions for single protein structures, and has recently been extended to genome-wide analysis of bacteria, identifying novel interactions in the co-evolution between resistance, virulence and core genome elements. However, earlier computational DCA methods have not been scalable to enable model fitting simultaneously to 10^4^–10^5^ polymorphisms, representing the amount of core genomic variation observed in analyses of many bacterial species. Here, we introduce a novel inference method (SuperDCA) that employs a new scoring principle, efficient parallelization, optimization and filtering on phylogenetic information to achieve scalability for up to 10^5^ polymorphisms. Using two large population samples of *Streptococcus pneumoniae*, we demonstrate the ability of SuperDCA to make additional significant biological findings about this major human pathogen. We also show that our method can uncover signals of selection that are not detectable by genome-wide association analysis, even though our analysis does not require phenotypic measurements. SuperDCA, thus, holds considerable potential in building understanding about numerous organisms at a systems biological level.

## Data Summary

1. Sequencing reads for the Maela population have been deposited in the National Center for Biotechnology Information Sequencing Read Archive (SRA) under study numbers ERP000435, ERP000483, ERP000485, ERP000487, ERP000598 and ERP000599.

2. Multiple sequence alignment for the Maela population is available from the Dryad Digital Repository: http://dx.doi.org/10.5061/dryad.gd14g.

3. Sequencing reads for the Massachusetts population have been deposited in the European Nucleotide Archive (ENA) under project number ERP000809, with accession numbers ERR065287 – ERR129216 (url - https://www.ebi.ac.uk/ena/data/view/PRJEB2632).

**Impact Statement**

The potential for genome-wide modelling of epistasis has recently surfaced given the possibility of sequencing densely sampled populations and the emerging families of statistical interaction models. Here, we introduce a novel and efficient inference method (SuperDCA) that enables analysis of genome alignments with up to 10^5^ polymorphisms on standard computational architectures. Using two large population samples of *Streptococcus pneumoniae*, we demonstrate the ability of SuperDCA to make additional significant biological findings about this major human pathogen. The genome-wide epistasis study (GWES) approach holds considerable potential in building understanding about numerous organisms at a systems biological level.

## Introduction

Direct coupling analysis (DCA) emerged less than a decade ago and has opened up a new direction of biological research by demonstrating that large population-based protein sequence analysis can be leveraged to make accurate predictions about protein structure [1–7]. DCA has been successfully extended to predict secondary and tertiary RNA structure [8], synergistic effects on fitness of mutations in the *Escherichia coli* lactamase TEM-1 [9], the fitness landscapes of human immunodeficiency virus proteins [10], and mutation effects from sequence co-variation [11], and to genome-wide epistasis analysis for bacterial population genomics [12]. Our focus here is to significantly extend the applicability of DCA methodology by enabling scalable inference for two orders of magnitude larger than previously modelled dimensionality of sequence positions.

Direct calculation of probabilities for the Potts models employed in DCA is intractable due to the form of the normalizing constant of the model distribution. Albeit Markov chain Monte Carlo (MCMC) -based sampling methods can be employed to obtain maximum-likelihood estimators, various weaker criteria or approximations have often been used to derive estimators of the model parameters. Notably, maximum pseudolikelihood is a statistically consistent inference method that has typically outperformed variational methods [13], such as the mean-field estimator [14]. The different software implementations based on regularized maximum pseudolikelihood for DCA applications (plmDCA) [3, 14–17] have been designed for protein sequences with the maximum length of 1000–2000 amino acids.

To enable use of plmDCA at the whole-genome level, with the order of 10^5^ polymorphisms in a bacterial genome, Skwark *et al*. [12] stratified a genome into non-overlapping windows and sampled randomly one single nucleotide polymorphism (SNP) from each window to form haplotypes of approximately 1500 sequence positions, on which the plmDCA implementation by Ekeberg *et al*. [15] could be directly applied. They then used a large number of repeated random sampling of positions from the stratified genome to aggregate information about interactions between polymorphisms across the genome. While this approach was demonstrated to successfully capture both known and novel interactions, it remains very computationally intensive and may still leave important interactions undiscovered, as only a fraction of all possible combinations of interactions will be covered even when using large numbers of repeated samples. It is also a hybrid method that does not fully implement global model learning, which is a conceptually central point of DCA. To avoid these problems, here, we introduce a method termed SuperDCA, which can perform inference simultaneously for all SNP positions in a much higher dimension. These advances are based on a new computational architecture exploiting efficient parallelization and optimization to achieve scalability for up to 10^5^ polymorphisms. In addition to being significantly faster with more modest computational resources, we also show that the global inference with SuperDCA allows the discovery of previously undetected epistatic interactions that inform our understanding of bacterial biology related to survival of the pneumococcus at lower temperatures. SuperDCA is freely available from https://github.com/santeripuranen/SuperDCA.

## Methods

### Data pre-processing

Bi- or tri-allelic loci with a minor-allele frequency (MAF) greater than 1 % were included in the analysis, provided that gap frequency was less than 15 %. Gaps were not counted as alleles in the frequency calculations. To facilitate direct comparison with previous results [12], a separate dataset was prepared from the Maela input alignment using otherwise the same filtering rules, but for bi-allelic loci only. Filtering of 305 245 SNPs in total resulted in two Maela input datasets for SuperDCA containing 94 028 SNPs and 3042 samples using the former rules, and 81 045 SNPs and 3145 samples using the latter rules. A subset of 103 samples containing mostly low-quality reads were included in the data in the previous study, but here were removed from the source alignment prior to locus pre-selection for our 94 028 SNP set. For the Massachusetts population, the first set of filtering criteria resulted in 78 733 SNPs and 670 samples.

### Hardware and inference details

Parameter inference was performed using a single 20-core HP SL230s G8 compute node with dual Xeon E5 2680 v2 CPUs and 256 GB of DDR3-1667 RAM. Total wall clock run times were 186 h (Maela with 94 028 SNPs), 167 h (Maela with 81 045 SNPs) and 39 h (Massachusetts with 78 733 SNPs), including file I/O, pre-filtering and parameter inference. Weights correcting for the population structure, regularization and choice of hyper-parameters were calculated exactly as in the genomeDCA method [12]. Coupling estimates for the three data sets that exceeded the cut-off described below are provided as Tables S1–S3 (available with the online version of this article) at https://github.com/santeripuranen/SuperDCA/MGen_2018_Tables_S1-S3/.

### Prediction cut-off

**Fig. 1.**
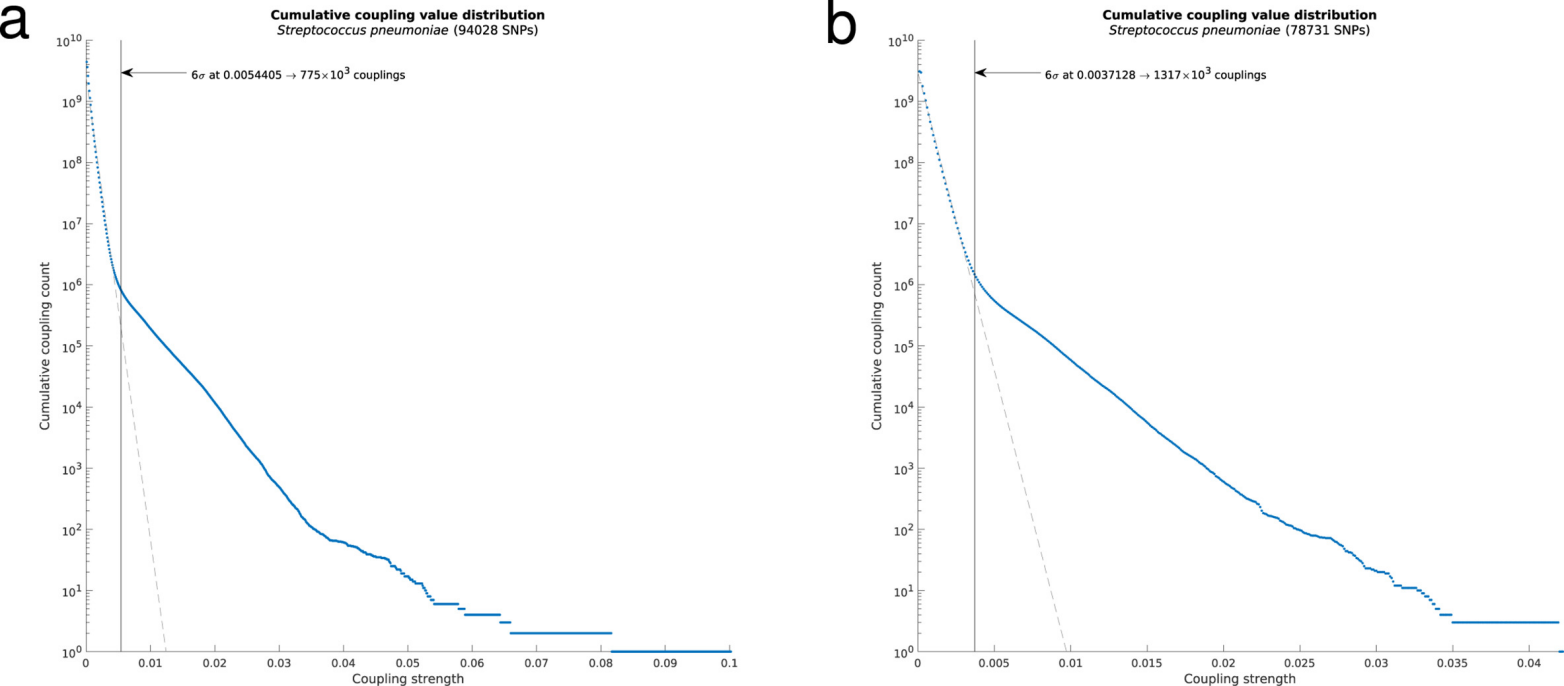
log histograms of the cumulative distributions of estimated between-site couplings for Maela (left) and Massachusetts (right) populations. The thresholds indicate the learned boundary between negligible and moderate to strong couplings.

The Potts models inferred in DCA are heavily over-parametrized. In protein contact applications, the benchmark number of parameters is typically in the millions (number of residue pairs times *q*^2^, where *q=*21), while the number of samples varies typically from thousands to hundreds of thousands. Therefore, only a small fraction of largest predictions is retained, commonly in the order of hundreds. For the present and future applications to whole-genome data, it is of more relevance to deliver a set of predictions at a pre-determined level of deviance from zero. An earlier approach using deviations from an extreme value theory distribution (Gumbel distributions) [12] was not applicable in the present set-up, since we are not only sampling the tail of the coupling coefficients but estimate couplings for all possible pairs of SNPs. As shown in Fig. 1, a semi-logarithmic cumulative distribution plot provides a computationally straightforward way to assess whether a particular coupling represents only random fluctuation near zero. The null distribution theory developed in Xu *et al*. provides a strong motivation for using the linear part of the distribution near the origin as representation of the noise level signals [18]. To obtain a threshold, we first performed a systematic scan over the histogram bins to fit a two-component linear spline function to the cumulative distribution. The standard deviation of the null couplings was then estimated using the part of the distribution between zero and the breakpoint. Similar to the Gumbel fit deviance level used by Skwark *et al*. [12], we then excluded all couplings that were less than six standard deviations away from the linear trend from further analysis. Fig. 1 illustrates that this procedure effectively filters out the vast majority of all possible couplings as noise, and allows the downstream analysis to focus on the relevant signals.

### Phylogenetic ranking of estimated couplings

By default, SuperDCA includes gaps as a state in the Potts model if they are found in the alignment at sites fulfilling the SNP pre-filtering criteria. Some gaps can be considered informative, representing indels, while some simply relate to sites that are difficult to sequence. Hence, some strong gap-induced couplings can represent lower-quality sequence data instead of true between-site interactions, and they should be automatically de-emphasized to better enable assessment of the biological meaning of the inferred couplings. Additionally, from a biological perspective, strong couplings that are signals of convergent evolution are the most interesting candidates for closer examination. To highlight the couplings where co-selection pressure has more likely repeatedly affected several minor allele combinations across the population, the SuperDCA coupling estimates are by default re-ranked using a combination of the three criteria described below, in addition to the actual value of the coupling.

Let *C* be a set of estimated couplings and $c_{i}=\left[c_{i1},c_{i2}\right]\in C$ a pair of SNP loci represented by their genome position indices. Let $y_{b}=\left[s_{1}^{\left(b\right)},\cdots ,s_{N}^{\left(b\right)}\right]$ be a haplotype over the *N* SNP loci. Further, $S_{i,1}$ is a set of haplotypes carrying a minor allele at locus $c_{i1}$ and $S_{i,2}$ a set of haplotypes with a minor allele at locus $c_{i2}$. The first phylogenetic ranking criterion is the minimum of the mean genome-wide Hamming distances of all pairs of isolates $y_{k},y_{l}\in S_{i,o},o=\left\{1,2\right\},k<l$, i.e. $d_{i}=min_{o}({\bar{d}}_{S_{i},o}(y_{k},y_{l})),i=1,...,\left|C\right|$ where ${\overset{-}{d}}_{S_{i,o}}\left(y_{k},y_{l}\right)=\frac{1}{|S_{i,o}|}\sum_{n=1}^{N}\left(s_{n}^{\left(k\right)}\neq s_{n}^{\left(l\right)}\right).$

Our second criterion is the normalized number of hierBAPS [19] clusters including isolates carrying the minor alleles at the two coupled loci, i.e. $a_{i}=\left|\left\{\beta_{b}|b\in \left(S_{i,1}\cap S_{i,2}\right)\right\}\right|,$ where $\beta_{b}$ is the designated hierBAPS cluster for haplotype *b*. Finally, the third criterion is the percentage of isolates where both SNP loci involved in a coupling had the minor allele, i.e. $m_{i}=\frac{1}{2}\left[\frac{\left|S_{i,1}\cap S_{i,2}\right|}{\left|S_{i,1}\right|}+\frac{\left|S_{i,1}\cap S_{i,2}\right|}{\left|S_{i,2}\right|}\right]$.

The above three criteria are normalized by $d_{i}^{norm}=\frac{d_{i}}{max_{i}(d_{i})},i=1,...,\left|C\right|,a_{i}^{norm}=a_{i}/max_{i}(a_{i})$ and $m_{i}^{norm}=m_{i}/max_{i}(m_{i})$ after which they are combined to a single ranking criterion $r_{i}=d_{i}^{norm}+a_{i}^{norm}+m_{i}^{norm}$ having a maximum value of three and a minimum equal to zero. Large values emphasize cases where both minor alleles at coupled loci are simultaneously widely distributed across the population. In cases where gaps at any two loci are phylogenetically spread in the population and would have led to a large estimated coupling values, they are still de-emphasized since they are not counted as minor alleles. The above criteria are derived by normalizing the individual coupling re-ranking measures developed by Skwark *et al*. [12]. The hierBAPS clusterings were obtained from the original publications introducing genome sequences for the Maela and Massachusetts populations [20, 21].

### Mutual information (MI) calculations

MI is an information theoretic measure of pairwise dependence between two variables calculated from the joint distribution over the variables. In the standard approach, the joint probabilities are estimated by the relative frequencies corresponding to maximum-likelihood estimates. Previous research has shown that a Bayesian estimator is more stable than the standard approach in terms of estimating the closely related concept of Shannon entropy from small samples [22]. Since we are dealing with relatively small efficient sample sizes, we used a corresponding Bayesian MI estimator in which a Dirichlet prior is put on the joint distribution and the estimator is defined as the expected value of the posterior density over MI given the data [23]. We defined the Dirichlet hyperparameters α by setting α=1/*K*, where *K* is the number of joint outcomes (Perks’ prior). To adjust for the population structure in the sample, we use the same re-weighting scheme as was applied in our SuperDCA inference with a similarity threshold of 0.90. Finally, to remove the influence of gap–gap interactions, we did not include sequences for which either of the two considered loci had a gap value.

### Genome-wide association study (GWAS) for the seasonality phenotype

We coded season as a binary variable based on whether isolates were acquired during the winter or the summer. We then tested 123 791 SNPs passing simple frequency filtering (>1 % MAF) for association with this variable using SEER [24], which performs a logistic regression at every SNP. We used the first three multi-dimensional scaling components of the pairwise distance matrix as fixed effects to control for population structure [24].

### Structural analyses

Crystal structures of *Streptococcus pneumoniae* penicillin-binding proteins (PBPs) with the following IDs, 2WAF (Pbp2b), 1QMF and 1RP5 (Pbp2x), were retrieved from the Protein Data Bank [25] (www.rcsb.org; accession date January 8 2016) and visualized in the PyMOL Molecular Graphics System, version 1.8.4.0 (Schrödinger). A chimera of 1QMF (chain A residues 257–618) and 1RP5 (chain A residues 64–256 and 619–750; missing sidechain atoms of E721 were reconstructed) was used for visualizing Pbp2x.

## Results

### Results of SuperDCA and comparison with genomeDCA

The Potts model for genome-wide epistasis analysis was fitted to two largest existing pneumococcal population data sets using the SuperDCA method: the Maela [12, 20] and Massachusetts populations [21]. Two variants of the Maela population data were considered: one with only bi-allelic SNPs (81 045 loci), filtered as in Skwark *et al*. [12] in order to maintain compatibility for comparison of the results, and the second with no restriction to bi-allelic SNP sites (94 028 loci; Methods). For Massachusetts, 78 731 SNP loci were analysed (Methods). Fig. 1 shows the cumulative distributions of the estimated coupling strengths between SNP sites for the Maela and Massachusetts populations. In both cases, a vast majority of the couplings were of negligible magnitude and could be discarded from further detailed investigation using the thresholds shown in Fig. 1 (Methods).

Fig. S1 shows the overlap between the predicted genomeDCA and SuperDCA links on a gene level for the Maela population. SuperDCA replicated the previously identified links between PBP gene pairs, as well as the network containing the *smc* gene. In contrast, SuperDCA did not identify significant links between *pspA*, *divIVA* and the triplet upstream of *ply*, SPN23F19480–19500. In the simultaneous analysis, which is not affected by chromosome stratification and random sampling of positions, the respective couplings no longer clearly deviated from the background dependence distribution, which is considerably wider for SuperDCA than for genomeDCA. This was illustrated by a closer examination of the pairwise MI values (for further details see Methods) between the SNP loci in *pspA*, *divIVA* and SPN23F19480–19500. The few stronger pairwise dependencies between the three genes disappear when all SNP loci are considered simultaneously. However, we wish to emphasize that these differences should not be over-interpreted and that further general examination of the pros and cons of simultaneous versus pairwise analyses will be a fruitful topic for future research.

### Epistasis in the PBPs

**Fig. 2.**
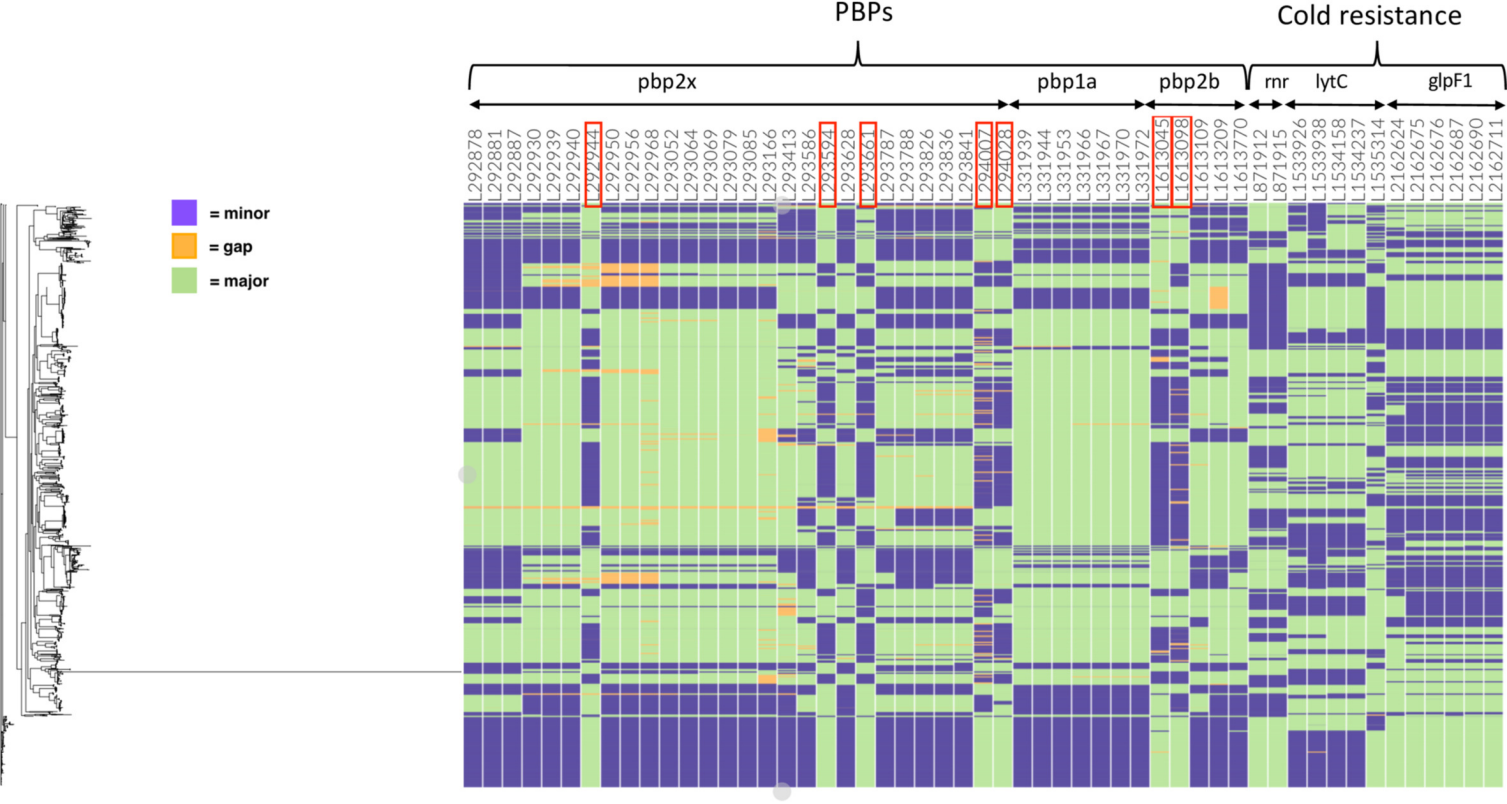
Maela population distribution of alleles at top ranked coupled SNP sites. The estimated genome-wide maximum-likelihood phylogeny is shown on the left. Each column is labelled by the genome position, gene name and a corresponding functional categorization. Columns marked by red rectangles indicate coupled sites in *pbp2x* and *pbp2b* that have a reversed minor/major allele distribution compared with the remaining displayed SNPs in the same genes.

Since the bulk of the biological signal of between-site variation dependence presented in Fig. 1 is due to linkage disequilibrium (LD) between sites in close proximity (Fig. S2), we used a refined version of the phylogenetic ranking of the couplings (Tables S1–S3; Methods) to focus on the strongest candidates of co-selected loci. Fig. 2 shows two sets of SNP loci that are involved in the top-ranking couplings in the Maela population, alongside with the phylogenetic distribution of the alleles. The very top-ranking couplings are between sites in the three PBPs, as discovered in the earlier epistasis analysis that stratified the genome into non-overlapping windows and used the Potts model for sampled subsets of loci to reduce dimensionality [12].

**Fig. 3.**
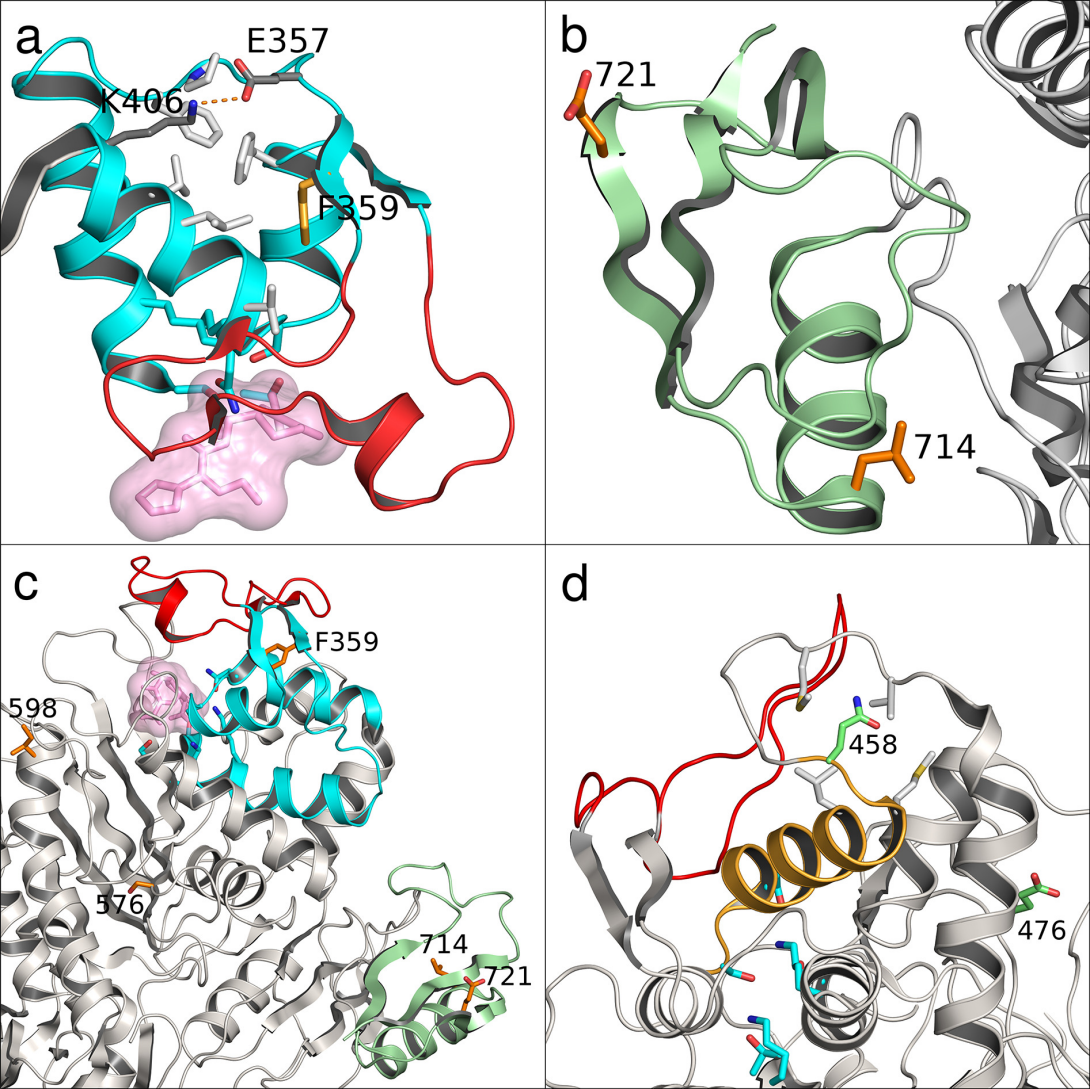
Structural mapping of the Pbp2x (a–c) and Pbp2b (d) positions marked in Fig. 2. The panels show the transpeptidase domains of each PBP with active site residues shown in cyan and positions marked in Fig. 2 as sticks in orange or green. (a) depicts a structure-stabilizing cluster of conserved hydrophobic residues (light grey sticks) and charge interaction (dark grey) in a region proximal to (cyan cartoon) the Pbp2x active site (with bound inhibitory antibiotic as pink space-filling volume) and a mobile loop (red cartoon) covering the active site. (b) depicts the PASTA-2 domain essential for divisome complex function (green cartoon) with the bulk of the protein to the right (grey cartoon). (c) shows an overview of the Pbp2x transpeptidase domain coloured as in the detail views in (a) and (b). (d) depicts the Pbp2b transpeptidase domain region proximal to the active site with a helix (orange cartoon) mechanically connecting the active site to the 'top' of the protein. An adjacent mobile loop covering the active site is shown in red.

Fig. 2 reveals a particular pattern of dependence between PBP mutations that adds significant biological information to the earlier findings [12]. The SNP positions marked by red rectangles in Fig. 2 have an approximately reversed distribution of minor/major alleles in the population, which may reflect fitness differences regarding co-evolution of emerging mutations. In Pbp2x, the first marked position (codon position 359) corresponds to a synonymous mutation encoding amino acid phenylalanine, part of a conserved cluster of hydrophobic residues (Fig. 3a, c) consisting of F353, P354, F393, L402, L403 and the E357 to K406 charge interaction located at the upper part of the transpeptidase domain near the active site. This cluster of residues likely has a role in maintaining structural integrity in this region (marked with cyan), as it is positioned next to the more mobile loop (marked with red) at residue positions 362–383 that partially covers the active site. Selection pressure seems to act in favour of the phenylalanine phenotype, since the genotype space clearly is explored here, and switching the phenotype to the similarly sized and hydrophobic (but in contrast to phenylalanine non-aromatic) residues leucine or isoleucine would only require a single non-synonymous mutation.

The second and third mutations (codon position 576, N/S/H amino acid changes; codon position 598, I/V amino acid changes) are conservative changes (Fig. 3d) that may remotely affect the active site geometry or substrate association/dissociation kinetics, possibly as a compensatory mechanism for changes elsewhere. Active-site reshaping is an established cause of β-lactam resistance in *S. pneumoniae*, where the involved polymorphisms can appear quite subtle at first sight. Our LD adjusted coupling scores indicate a very strong coupling between genome positions 294 028/293 661 in *pbp2x* and 1 613 045/1 613 098 in *pbp2b*. The fourth and fifth mutations (codon position 714, conserved L amino acid; codon position 721, E/Q amino acid change) are located in the PASTA-2 domain (Fig. 3b; marked with green). The Q721 variant is prevalent in β-lactam susceptible and E721 in non-susceptible isolates. PASTA (PBP and serine/threonine kinase associated) domains typically bind β-lactams; however, a direct mechanistic role for 721 in β-lactam resistance seems unlikely due to the structural position facing away from the protein core region. Rather, 721 is more likely to be involved in divisome complex formation and functions in a way that supports bacterial resilience in the presence of antibiotics; Pbp2x and the PASTA domains therein are essential for bacterial division [26, 27]. The characteristics and placement of L714 and the fact that all polymorphisms at this site are synonymous, point to a role in assuring structural integrity rather than in direct β-lactam interaction.

In Pbp2b, the second marked position (codon position 458, D/N amino acid change) is located such that it may affect the active site in a mechanistic way via two distinct routes, either by indirectly modifying stability of the loop region (marked with red) proximal to the active site or by slightly affecting the geometry of active site residues through the helix from 445 to 456 (marked with orange) directly connected to active site residues N445 and S443. The first marked position in Pbp2b (codon position 476, G/E amino acid change) is spatially separated from 458. Although glycine at this site is more prevalent in β-lactam non-susceptible- and glutamic acid in susceptible isolates, the potential role of the residue at this position in resistance remains unclear and would be a target for further experimental work.

**Fig. 4.**
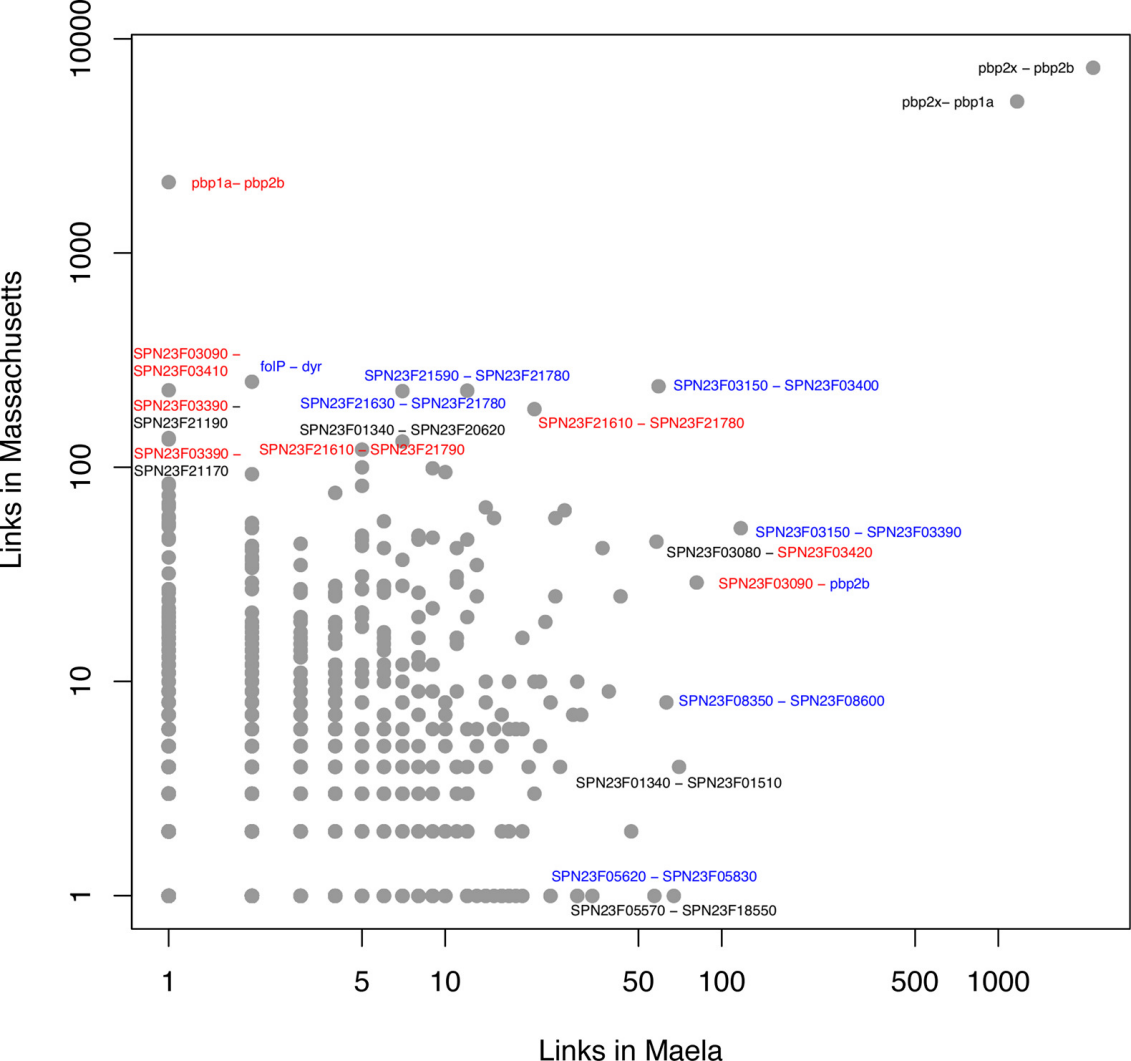
Overlap of estimated SNP interactions between the Maela and Massachusetts populations. Each dot represents an estimated link (interaction) between two coding sequences (CDSs), the blue CDSs are involved in antibiotic resistance, and the red CDSs are in close proximity to antibiotic resistance loci. Grey dots represent other functional categories not displayed here explicitly for visual clarity. Both axes are on a log scale and the values represent numbers of links in each CDS pair.

Fig. 4 shows a clear overlap between the Maela and Massachusetts populations in terms of identified links between genes involved in antibiotic resistance. For the two PBP-encoding gene pairs *pbp2x-pbp2b* and *pbp2x-pbp1a*, the numbers of strong links between SNPs are large in both populations. For the pair *pbp1a-pbp2b*, there is a pronounced asymmetry in this respect, such that the Massachusetts population harbours a large number of links, whereas there are only very few in Maela. The latter observation is in line with the findings by Skwark *et al*. [12], which indicated that most interactions found between the PBP-encoding genes were between *pbp2x-pbp2b* and *pbp2x-pbp1a*. The fact that the Massachusetts population clearly deviates from this suggests that the co-evolution of PBPs may follow a non-congruent route in different populations. In the case of Massachusetts versus Maela, this may be a consequence of markedly different serotype distribution in the two populations, or other ecological constraints such as the varying selection pressure from different β-lactam antibiotic usage. In the Maela population, β-lactam prescriptions were almost exclusively amoxicillin, whereas in the Massachusetts population the paediatric prescription practice is likely to have been considerably more varied. Similar to the asymmetry of the extent of *pbp1a-pbp2b* couplings, the reverse allele distribution pattern discussed previously for Maela was not observed in the Massachusetts population. Given these differences, our results suggest that the co-selective pressure on PBP-encoding gene polymorphisms acts differently depending on the type of the β-lactams used in the population, warranting further experimental work to elucidate the mechanistic role of the coupled variations.

### Epistasis in cold tolerance and transmission potential

The current analysis additionally highlights several important between-site dependencies not identified by genomeDCA, showing greater sensitivity for identifying putative epistatic interactions. Firstly, the highest ranked SuperDCA couplings included 20 links between cold-resistance-related genes exoribonuclease R (*rnr*), glyceroporin (*glpF1*) and lytic amidase C (*lytC*) (Fig. 2), the strongest of which was ranked 668. In total, among the 5000 highest ranked couplings, there were 2 links between *glpF1* and *rnr*, and 18 links between *glpF1* and *lytC.* GlpF1 is a transporter than imports glycerol and is involved in maintaining membrane fluidity with temperature changes [28]. The *glpF1* gene is at the 3′ end of its operon, with a tightly-folding BOX repeat at its distal end [29]. This would make the corresponding mRNA a potential target for Rnr, a cold shock response 3′→5′ exonuclease that degrades tightly-folded RNAs that might be misfolded at lowered temperatures. Hence, these interactions may be involved in tuning the expression of *glpF1* at lowered temperatures. Like GlpF1, LytC is involved in maintaining the cell surface at lower temperatures, as it is the cellular amidase specialized at degrading peptidoglycan at lower temperatures (30 °C, rather than 35–37 °C) [30].

**Fig. 5.**
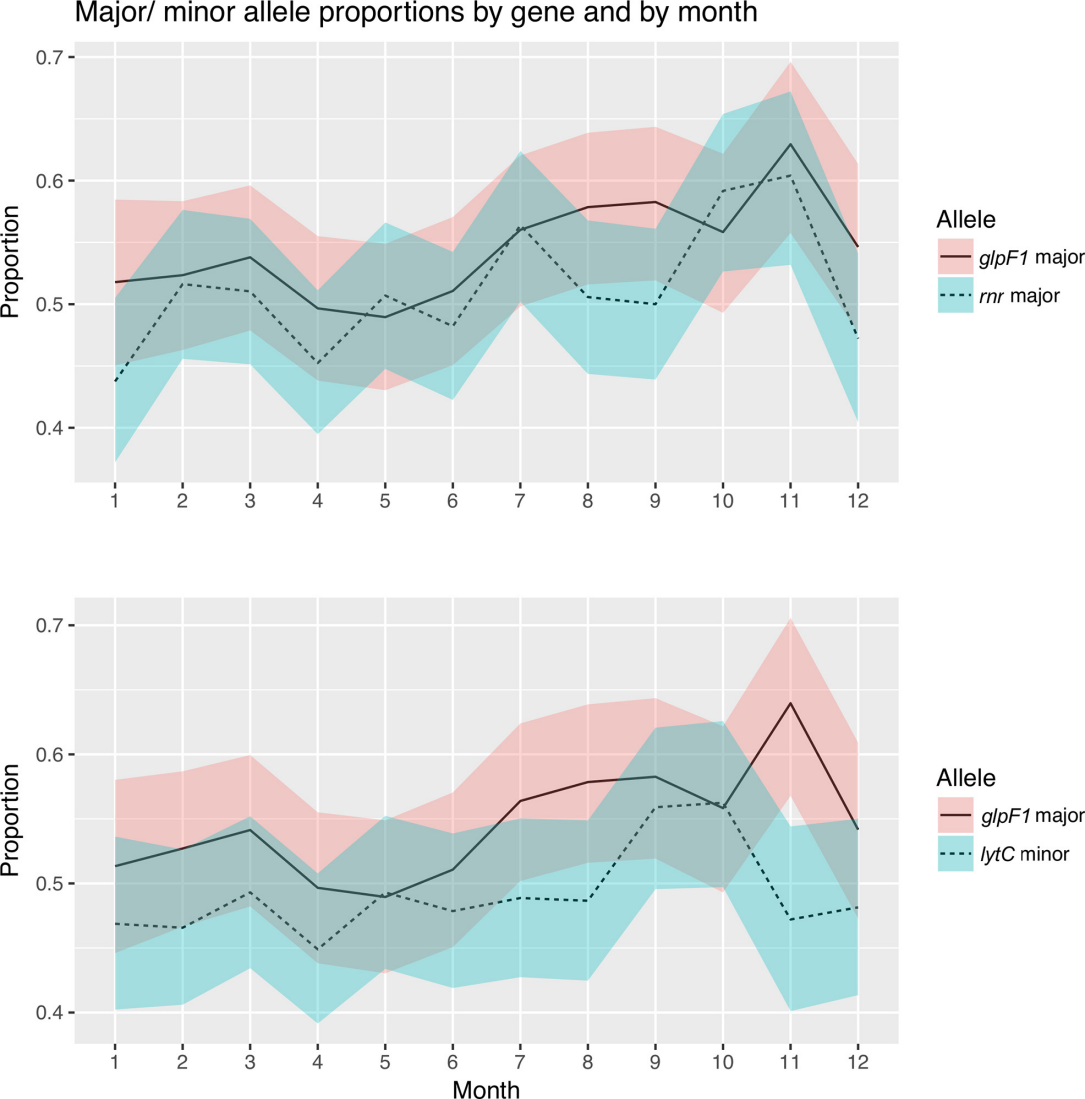
Seasonal variation of the allele frequencies for the two top cold-resistance couplings between *glpF1-rnr* and *glpF1-lytC* averaged over 3 years, 2007–2010. The shaded areas indicate 95 % confidence intervals.

Previous work has demonstrated a significant seasonality in the transmission dynamics for the Maela population, while carefully controlling for viral epidemics; the probability of the transmission being higher during the cold and dry winter months in comparison to warmer and more humid spring and summer months [31]. To examine whether the observed epistatic links related to survival at lower temperatures are connected with the seasonal transmission phenomenon, we examined the major allele frequencies and MAFs at the strongly linked cold-resistance loci according to months, averaged over the 3 years, 2007–2010, during which the data were sampled. Fig. 5 shows clear temporal signals in terms of when the isolates carrying the linked minor/major alleles were sampled. The temporal changes in allele frequencies for the strongest cold-resistance-related link between *glpF1* (position 2 162 687) and *rnr* (position 871 912), and also for the most strongly coupled sites between *lytC* (position *1 533 938*) and *glpF1* (position *2 162 676*), display a repetitive pattern of synchrony across years. In the first case, the proportion of major alleles in *glpF1* increases towards the end of the year, while in *rnr* the proportion of the minor alleles varies, being the dominant allele in January, April and December. In the second case, the pattern in *glpF1* remains the same, but the proportion of minor alleles in *lytC* increases towards the winter months.

These findings, combined with the earlier results on Maela hosts being more susceptible for transmission during the cold and dry winter months [31], suggest that the recurrent selective advantage related to increased cold tolerance to facilitate survival outside hosts has been sufficient to shape the variation in population allele frequencies. To investigate whether the selection pressure on cold-resistance genes could be discovered using a GWAS approach, we coded the phenotype of each sample as winter or summer depending on the sampling date (Methods). We then applied the SEER GWAS method to identify polymorphisms that explain the variation in the phenotype [24]. Fig. S3 shows the Manhattan plot of the SEER analysis based on the annotated reference genome. No clear association signal can be seen and the SNP loci within the cold-resistance genes are not associated with any markedly smaller *P* values than the level of background variation of the association signal.

No cold-resistance-related couplings were found among the top 5000 couplings in the Massachusetts population, which may represent the less variable environmental conditions to which children are exposed, and the sampling of isolates only during winter, rather than year round. In contrast, the Maela refugee camp conditions are such that the changes in selection exposure are more directly influential.

### Filtering on phylogenetic information

**Fig. 6.**
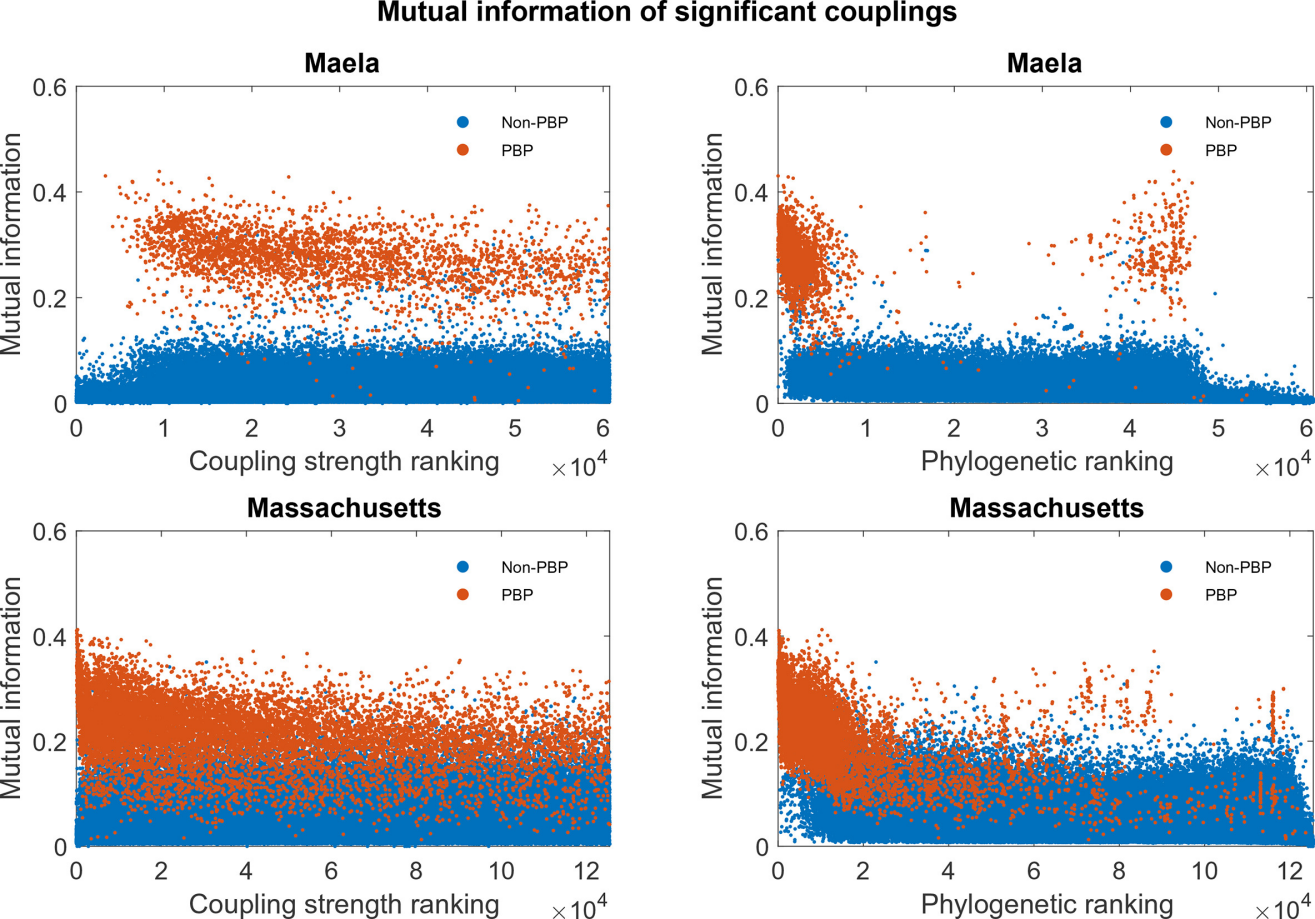
Estimated MI for 60 749 pairs of SNPs (Maela) and 125 469 pairs of SNPs (Massachusetts).

Inferred couplings from DCA typically have to be filtered to remove those that refer to trivial or non-informative dependencies. In the protein-structure applications, very strong couplings are inferred among close neighbours along the peptide backbone, and are usually removed after model fitting by a simple distance-based cut-off. A related issue is sampling bias, which for protein-structure applications has been handled by a reweighting applied to each sequence [1]. In bacterial sequence data produced from a sample taken from a small area over a limited period of time, a further issue is clonal inheritance; the meta-population is in a state of flux, and for a short window of time may not fully relax to the postulated Potts model of DCA. To compensate for this problem, we used a refined version (Methods) of the phylogenetic re-ranking of the coupling estimates introduced in Skwark *et al*. [12] To visualize its effect, we consider MI to characterize the strength of pairwise dependencies between SNP loci. MI is a widely used information theoretical measure of dependence between discrete-valued variables, and it has been a popular tool as part of bioinformatics methods for DNA sequence analysis [32–34]. Here, we use MI to characterize the strength of pairwise dependence between SNP loci as a function of their ranked estimated couplings alone, and a ranking based jointly on couplings and phylogenetic criteria. Fig. 6 shows the distribution of inferred MI values (Methods) for the two rankings in both the Maela and the Massachusetts population. The PBP-related couplings are nearly universally associated with higher MI values, indicating their tighter co-evolution despite the negligible level of background LD between the three PBP segments. The distributions of large MI values have a clear shift towards a higher rank for both Maela and Massachusetts populations, which succinctly demonstrates the usefulness of using a phylogenetic ranking of coupling estimates to highlight co-selected sites above the background LD. A comparison of MI distributions for PBP-related SNPs for the two populations revealed that Maela displays stronger dependencies between the PBP mutations than Massachusetts (Fig. S4).

### Performance improvements in SuperDCA

Overall, SuperDCA achieved an 18-fold effective performance increase over the earlier reference plmDCA implementation [15] on a single 20-core dual-socket compute node, enabling inference of 1.4×10^11^ parameters for a 94 028 SNP genome dataset in less than 8 days, instead of an estimated 170 days. This was achieved through multiple alterations to the central algorithm explained below. Let $\left(s_{1},s_{2}\ldots ,s_{N}\right)$ be a haplotype over *N* SNP loci, where each *s_i_* can take values from an alphabet with cardinality *q*. Typically, this cardinality varies between three (allelic states: minor/major/gap) and five (allelic states: A,C,G,T, gap). A Potts model assigns a probability distribution on such haplotypes defined by the following formula

$$P\left(s_{1},s_{2},\ldots ,s_{N}\right)=\frac{1}{Z}e^{E\left(s_{1},s_{2},\ldots ,s_{N}\right)}$$

where the normalizing constant *Z* is known as the partition function and the expression in the exponent is

$$E\left(s_{1},s_{2},\ldots ,s_{N}\right)=\sum_{i=1}^{N}\sum_{a=1}^{q}h_{i}\left(a\right)\delta_{s_{i},a}+\sum_{i,j=1}^{N}\sum_{a,b=1}^{q}J_{ij}\left(a,b\right)\delta_{s_{i},a}\delta_{s_{j},b}$$

In above $\delta_{x.y}$ represents the Kronecker delta function, which takes the value one if the arguments *x* and *y* are equal, and is otherwise zero. The linear terms are $h_{i}\left(a\right)\delta_{s_{i},a}$ for different SNP loci and their alleles. The coefficients $h_{i}\left(a\right)$ parametrize a deviation from the uniform allele distribution for each SNP, independently of the values of all the other variables. The quadratic terms are the matrix elements $J_{ij}\left(a,b\right)\delta_{s_{i},a}\delta_{s_{j},b}$ for different combinations of values of *i* and *j,* and *a* and *b*. The coefficients $J_{ij}\left(a,b\right)$, which are the *couplings* or *interactions* of pairs of SNPs, are defined as zero when the two indices *i* and *j* are equal. A coupling matrix with all elements equal to zero for non-identical locus index pairs implies that the alleles at these two loci are distributed independently in the population. Small positive values of the coupling matrix elements correspond to weak dependence between the SNP loci. In this paper, we have addressed the issues of gauge invariance and gauge fixing in the Potts model [1], as described previously [12, 15].

One of the major obstacles for using earlier plmDCA algorithms simultaneously on large numbers of SNPs without locus subset sampling is their large runtime memory requirements. plmDCA memory use is dominated by the storage of *q*^2^-dimensional parameter matrices $J_{ij}$, where *q* is the cardinality of the SNP state space (the maximum value being *q* = 5 when a gap/indel is included). $J_{ij}$ and $J_{ji}$ are needed simultaneously for calculating the pairwise coupling value, and since the elements are inferred row- or column-wise for all *i* (or *j*) at a time, a straightforward implementation of the algorithm necessitates simultaneous storage of all couplings in an *N*-by-*N* matrix *J*; therefore, storage is needed for *q*²(*N*²-*N*) scalar elements. The scoring of the estimated coupling matrices would then be calculated according to

$$J_{ij}=J_{ji}={\left\|\left(J_{ij}+J_{ji}\right)/2\right\|}_{F}$$

where *F* indicates the Frobenius norm. As an example, if a 10^5^ SNP genome alignment was characterized by 5-state alphabet and parameters stored in 64-bit floating point format, then the full interaction matrix *J* would require approximately 1.8 TB of memory, which is typically beyond the RAM available in state-of-the-art HPC (high-performance computing) cluster nodes. However, if the scoring of coupling values is instead calculated as

$$J_{ij}=J_{ji}=\left({\left\|J_{ij}\right\|}_{F}+{\left\|J_{ji}\right\|}_{F}\right)/2$$

then runtime storage requirements are reduced by a substantial factor and the intermediate storage requirements for our example would shrink to 74 GB, which is well in the feasible range for current HPC nodes. Fig. S5 illustrates numerically that the above two scoring approaches lead to insignificant numerical differences in practice. SuperDCA uses this finding as one of its key improvements of plmDCA.

Performance profiling analysis identified high memory requirements and poor cache utilization as a major bottleneck for the performance in earlier plmDCA implementations when applied to higher-dimensional data. Parallel execution scaling also suffered due to memory bandwidth starvation. The maximization step was performed by Ekeberg *et al*. [15] using L-BFGS gradient-based optimization. However, the objective function required repeated traversal through all input data and the full parameter vector, emphasizing the need for an efficient data structure. To remedy these issues, a space-efficient, block-wise ordered data structure with simple state-pattern dictionary and run-length encoded indexing strategy for the genome data and a cache-friendly blocked memory layout for parameters were developed for SuperDCA and implemented in C++ (Fig. S6). A particular design choice was made to restrict the maximum value of *q* to 4. The resulting data structure reduced runtime memory use for nucleotide alignments by more than fourfold compared with a typical dense data matrix representation. It also helped to reduce computing effort, improved processor cache utilization and enabled efficient utilization of SIMD vector instructions. The aggregate effect of these changes was an eightfold improvement in single-threaded performance. The reduced main memory bandwidth use also helped improve node-level scaling as we measure a strong scaling factor of >0.7 up to 20 cores. Figs S7–S9 illustrate the computational scalability aspects for SuperDCA compared with genomeDCA.

## Discussion

Production of natural population genomic sequence data is currently still exponentially accelerating, highlighting the need for statistical methods that can generate detailed hypotheses for further experimental work regarding loci likely to be important in shaping bacterial evolution. Genome-wide association analysis has for a decade been the major general tool for such purposes, and more recently, its applicability to bacteria has been also demonstrated [24, 35–37]. Skwark *et al*. [12] showed for the first time that statistical genome-wide modelling of joint SNP variation using DCA can uncover valuable information about co-evolutionary pressures on a large scale. This was done without relying on any phenotypic measurements, and using a hybrid scheme that does not fully employ the global model learning aspect of DCA. Here, we built upon this initial observation to develop DCA into a powerful tool that is applicable to a majority of the existing bacterial population genome data sets in a computationally scalable manner. The biological insights on the differential evolution of PBPs, and the cold-tolerance mechanisms, derived from the results of applying SuperDCA to two of the largest available pneumococcal genome data sets illustrate succinctly how such an approach could provide vital clues to the evolutionary processes under different ecological conditions in natural populations.

As the size of genome sequence data sets keeps growing, even our optimized parallel inference algorithm will eventually become too inefficient for practical purposes. Currently, the chosen data and algorithmic architecture work extremely effectively for up to around 10^5^ polymorphisms. As bacterial whole-genome alignments are typically of the order of 10^6^ sites, this should be sufficient for most population genomic studies. After this, the runtime will start to increase so rapidly that different computational strategies will be required for data sets including significantly more SNPs. Thus, an important topic for future research is to investigate how the Potts model inference can be performed in a reliable manner without resorting to a quadratic increase in the computational complexity as a function of the number of polymorphisms.

## Data bibliography

1. Chewapreecha, C., Harris, S. R., Croucher, N. J., Turner, C., Marttinen, P., *et al.* National Center for Biotechnology Information Sequencing Read Archive (SRA) accession numbers ERP000435, ERP000483, ERP000485, ERP000487, ERP000598 and ERP000599 (2014).
2. Skwark M. J., Croucher N. J., Puranen S., Chewapreecha C., Pesonen M., *et al.* Dryad Digital Repository: http://dx.doi.org/10.5061/dryad.gd14g (2017).
3. Croucher, N. J., Finkelstein, J. A., Pelton, S. I., Mitchell, P. K., Lee, G. M., *et al.* European Nucleotide Archive (ENA) accession number ERP000809 (2013).

## Supplementary Data

5325 Supplementary File 1
