# Supplementary material for: SuperDCA for genome-wide epistasis analysis
Source: Microb Genom. 2018 May 29;4(6):e000184. doi: 10.1099/mgen.0.000184 (PMC6096938; doi:10.1099/mgen.0.000184)
Supplement: Supplementary File 1 [file mgen-4-184-s001.pdf]

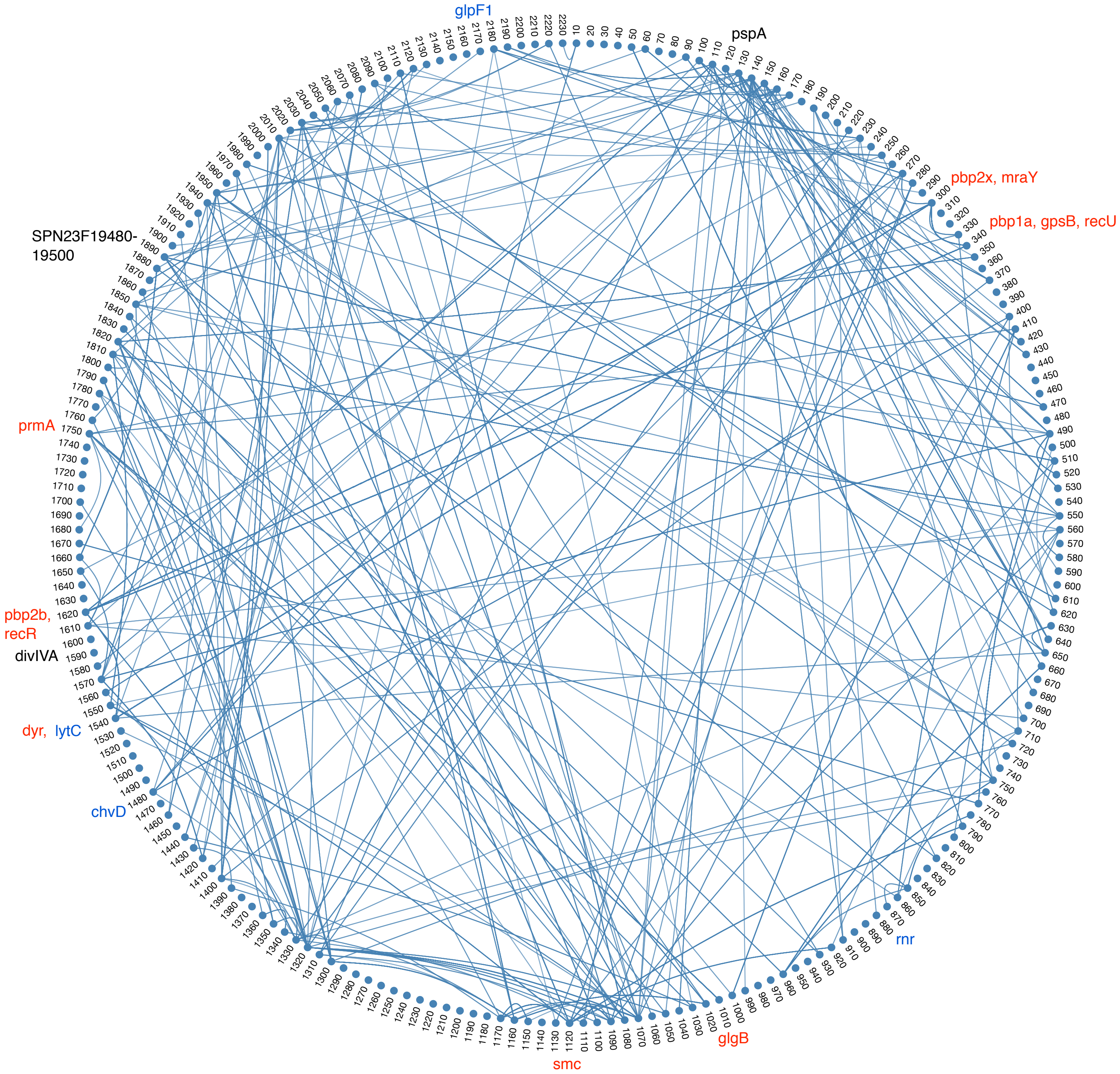

Supplementary Figure 1. Overlap of the predicted genomeDCA and SuperDCA couplings in Maela population.

Lines are plotted between genes having at least three SNPs linked from the same genes, both in genomeDCA and SuperDCA. This results in 274 overlapping interactions. The thickness of lines is proportional to the number of linked positions within the corresponding genes. Gene annotations shown outside the circle are centered at the positions of the corresponding genes. Red labels are given for genes linked tightly both with genomeDCA and SuperDCA, black labels for genes linked only with genomeDCA and blue labels for genes linked only with SuperDCA.

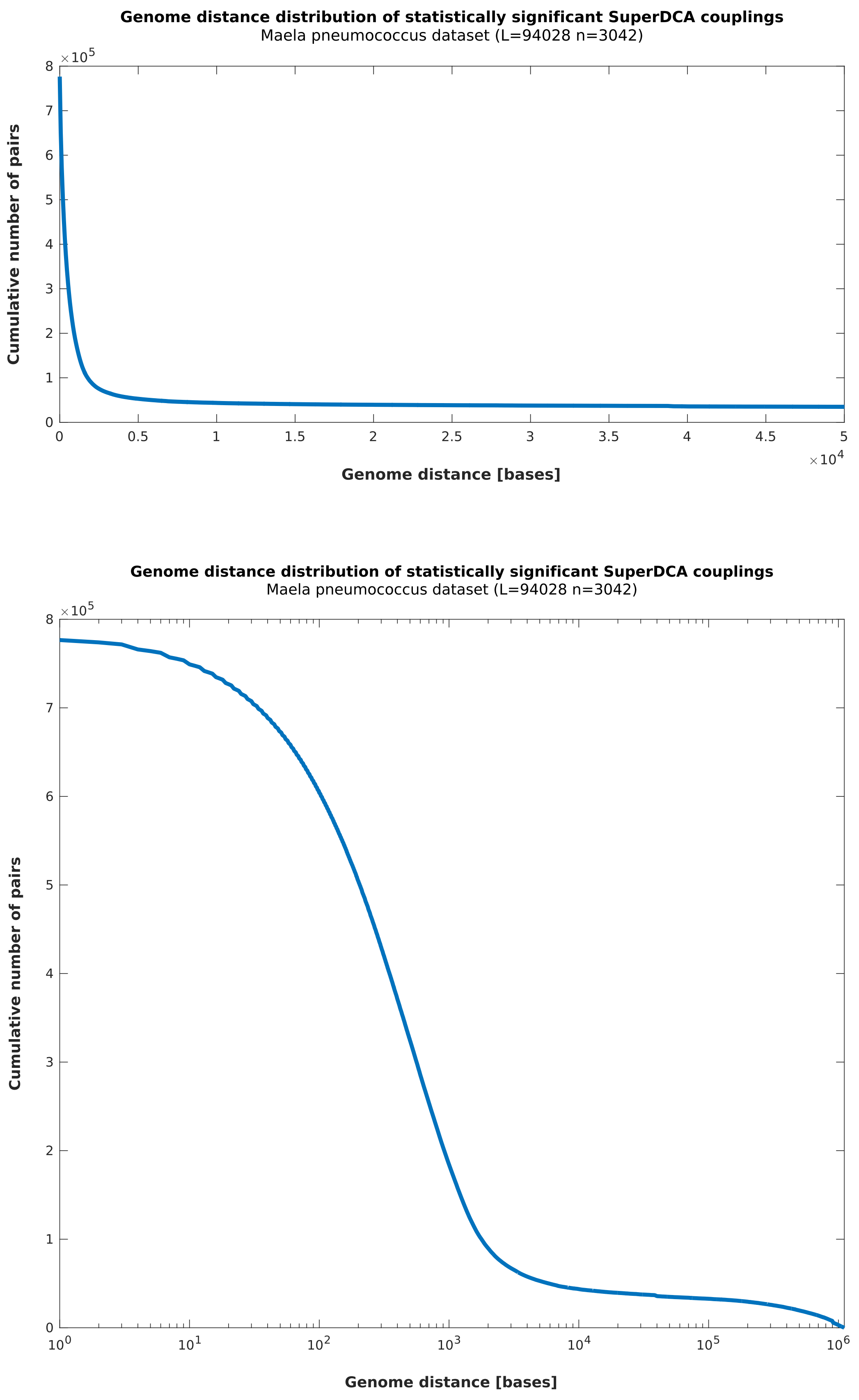

Supplementary Figure 2. Genome distance distribution of statistically significant SuperDCA couplings.

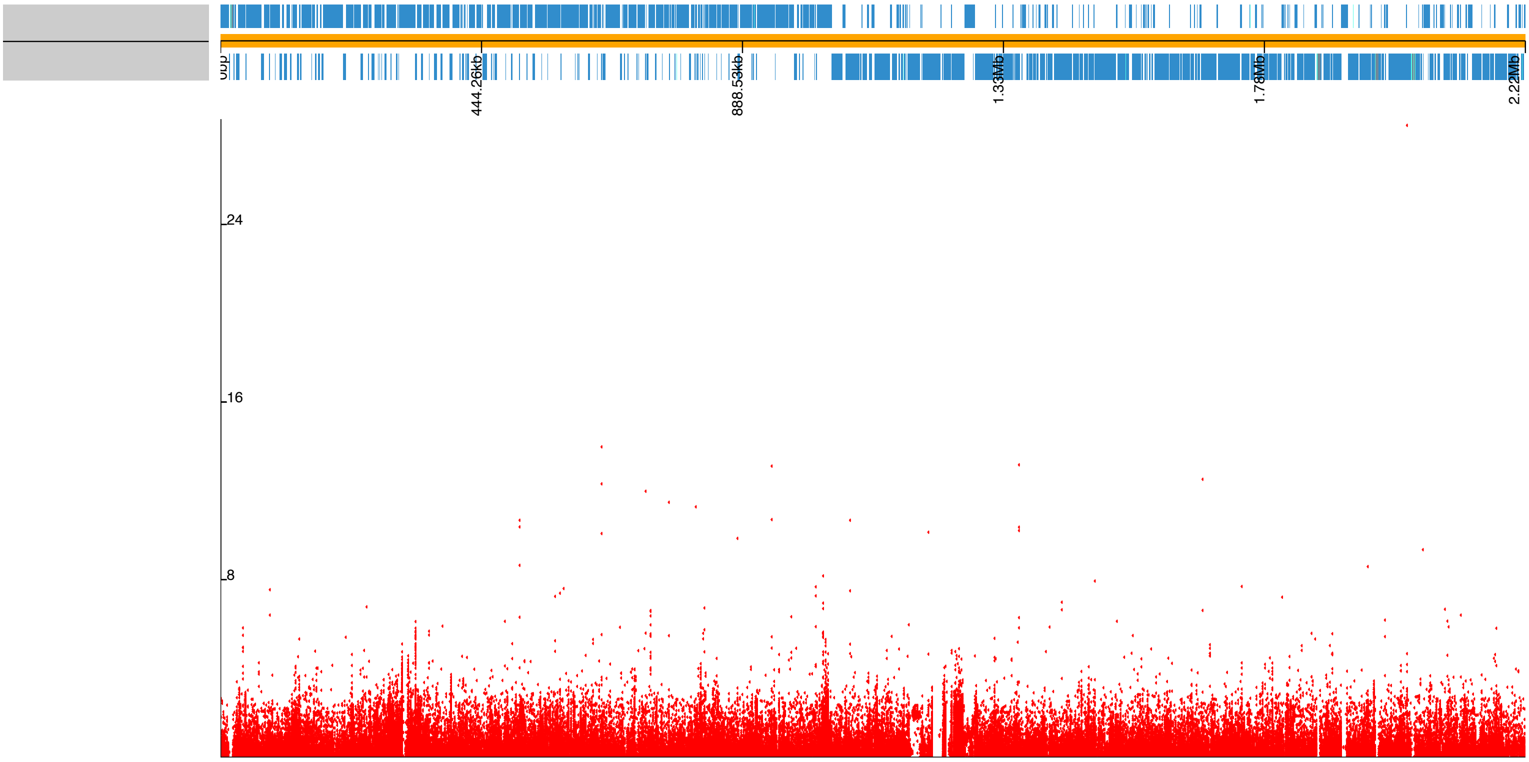

Supplementary Figure 3. SEER GWAS Manhattan plot of p-values for the winter/summer phenotype.

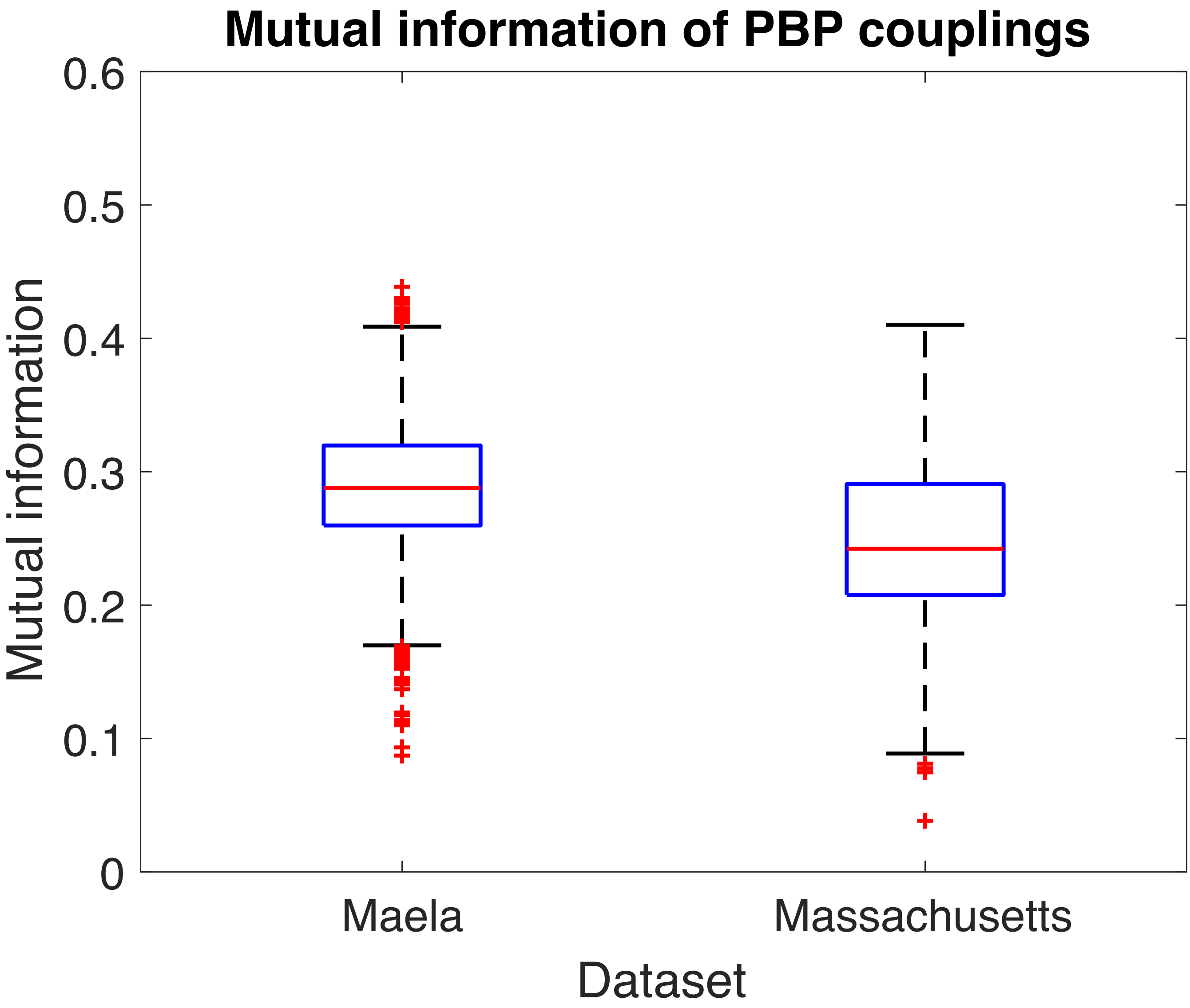

Supplementary Figure 4. Boxplots of MI value distributions for pairs of SNPs in two different PBP genes.

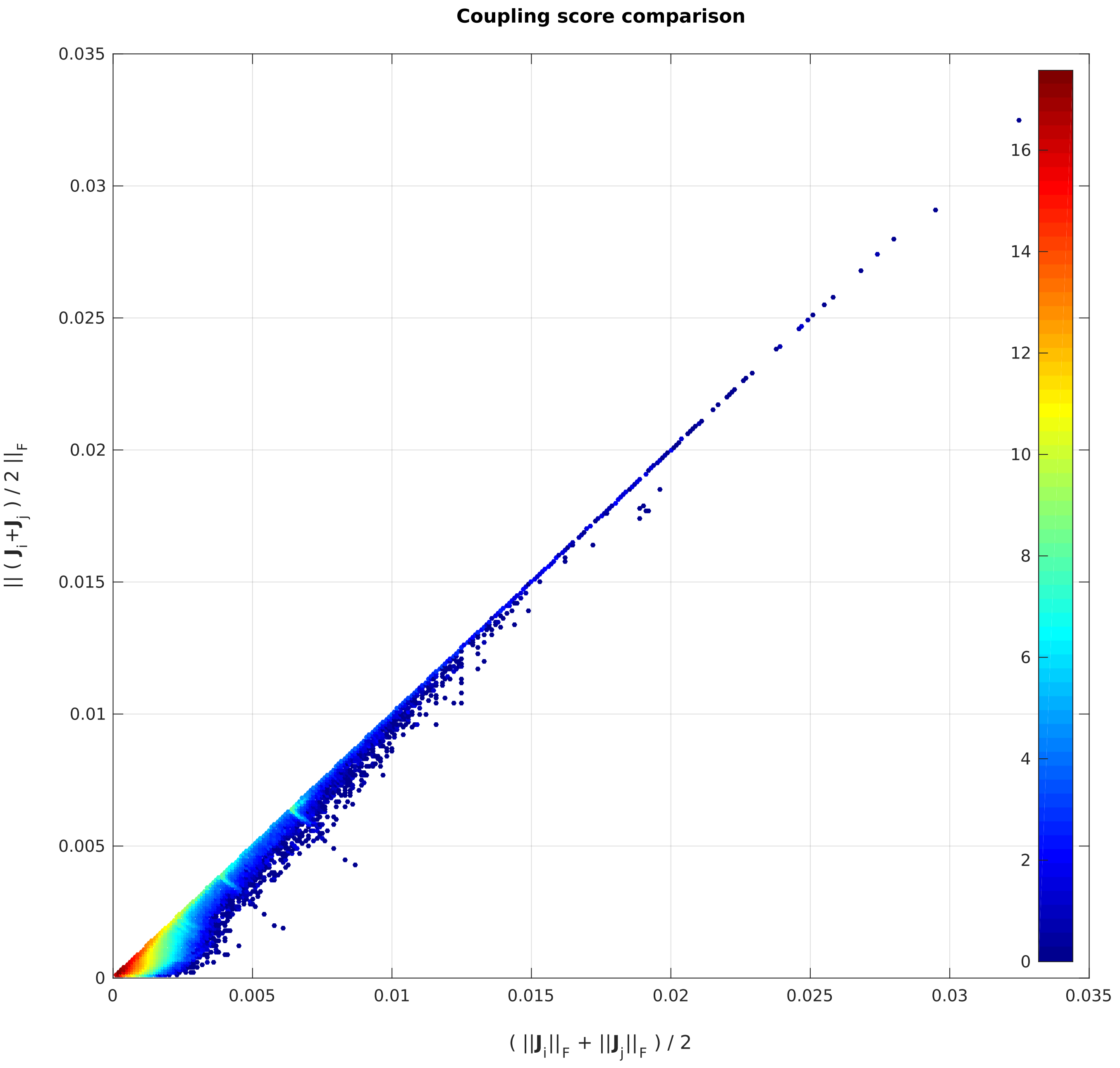

Supplementary Figure 5. Comparison of the norm-of-mean (vertical axis) versus the mean-of-norms (horizontal axis) summary score strategies.

Differences between the two are negligible for stronger, statistically significant coupling values and show more pronounced deviations only towards the sub-significant domain. The plot was calculated using a 25% uniformly random sample of loci from the 94028 SNP Maela dataset using the full data as background. Coloring marks coupling value count in log-scale.

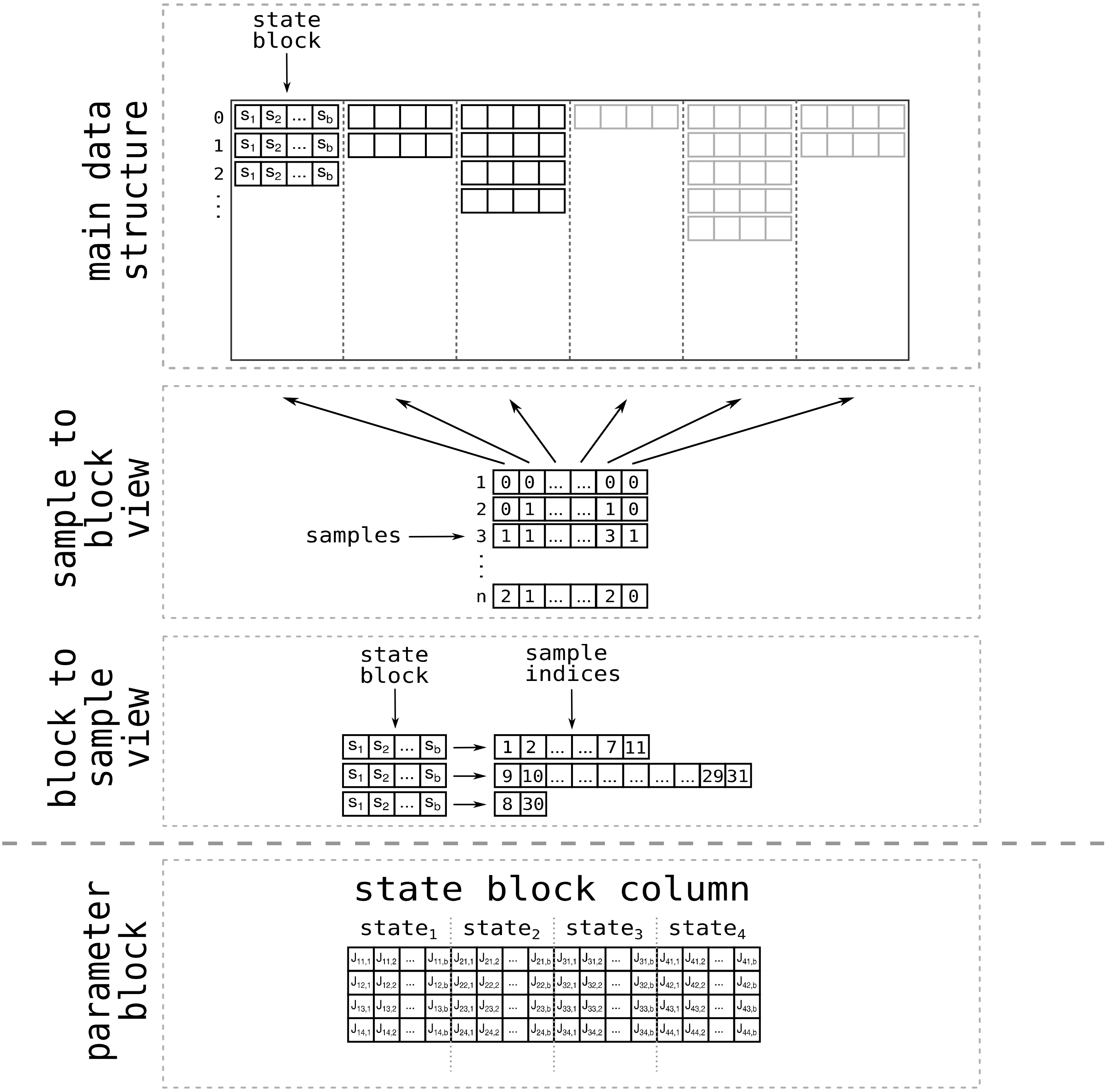

Supplementary Figure 6. Schematic drawings of the central data structures used in SuperDCA for storing input state data (nucleotide alignments) and the inferred parameters.

The input data matrix is stored such that samples (i.e. isolate genomes in our case) are ordered row-wise, with each sample divided into blocks of size b. Only column-wise unique blocks are stored. Block indices are stored for sample-oriented access to the data and sample indices for column- oriented access. Block index lists can optionally be run-length encoded, which leads to very significant space savings in particular when storing full-genome alignments with large regions of low column-wise variation. Index-lists for column- oriented access can similarly be collapsed for saving storage space when indices form contiguous (ascending) sequences. The inferred parameters are stored in blocked format such that all parameters relating to a particular column block in the input data are grouped together.

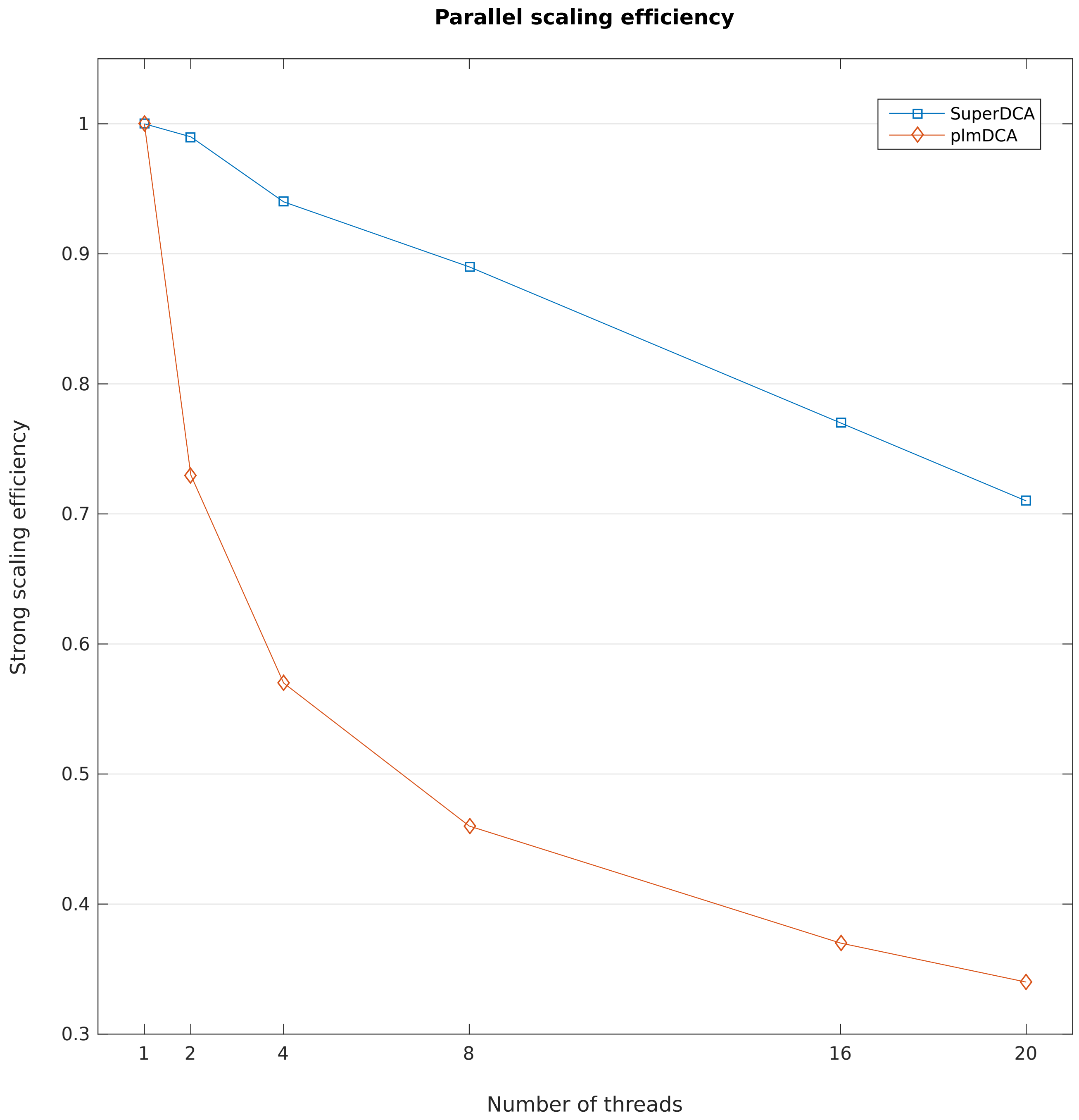

Supplementary Figure 7. Comparison of SuperDCA versus plmDCA parallel scaling efficiency.

SuperDCA (blue curve) shows markedly stronger scaling than plmDCA (red curve). The scaling numbers were obtained as a mean of three runs of three 2-permil uniformly random samples of loci (188 loci) from the 94028 SNP Maela dataset and using the full data as background. Inferred parameter storage was disabled in plmDCA for the purpose of benchmarking. All benchmarks were run on a single 20-core HP SL230s G8 compute node with dual Xeon E5 2680 v2 CPUs and 256GB of DDR3-1667 RAM.

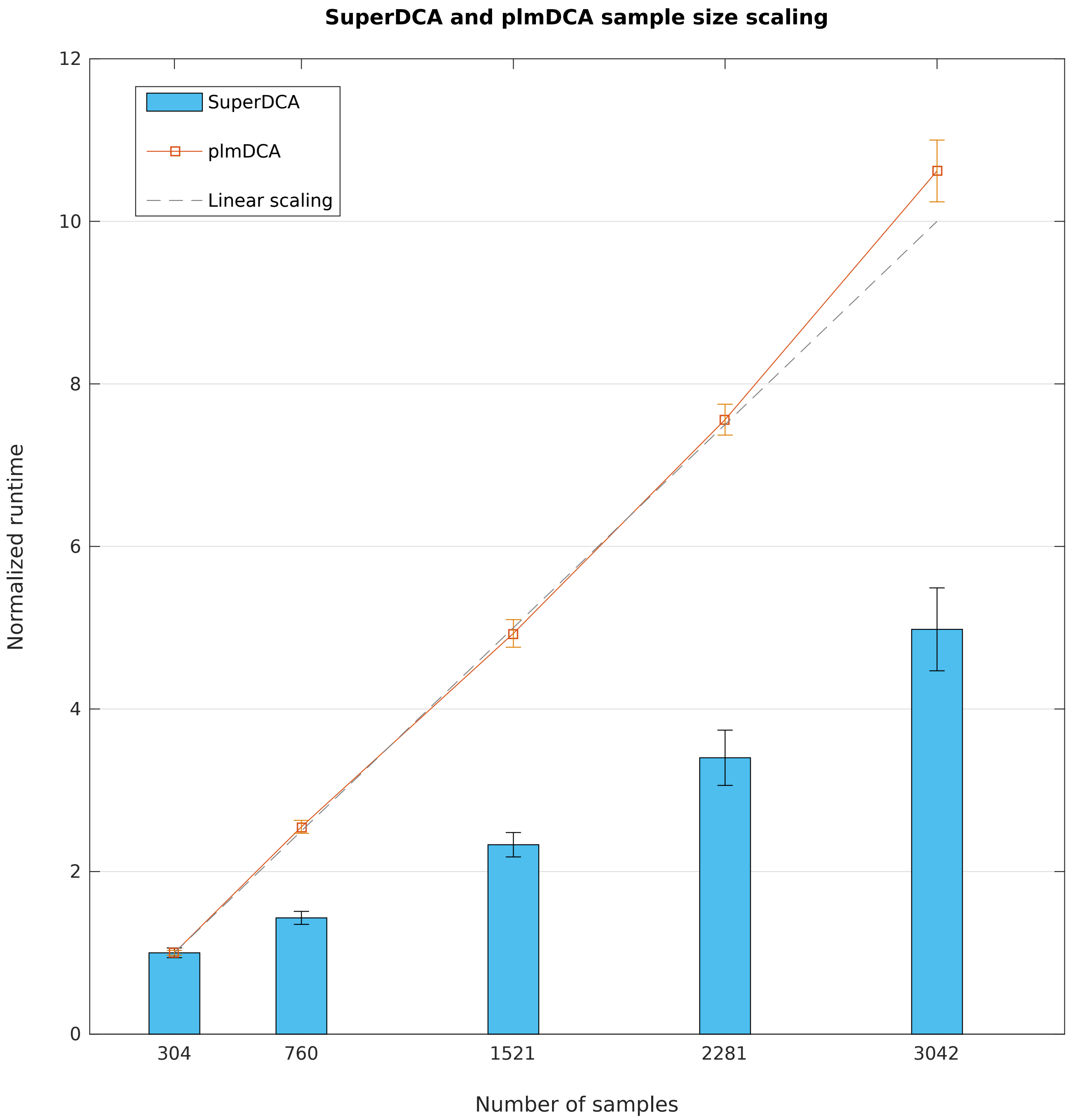

Supplementary Figure 8. Comparison of SuperDCA versus plmDCA sample size scaling.

The sample-compressing datastructure used in SuperDCA enables markedly stronger scaling (blue bars) with increasing sample size than plmDCA (red curve). The scaling numbers were obtained as a mean of 9 runs: three-by-three sets of runs using a uniformly random sample of sequences and run for three 2-permil uniformly random samples of loci (188 loci) from the 94028 SNP Maela dataset and using the full data as background. See caption of Fig. S7 for details of benchmark hardware.

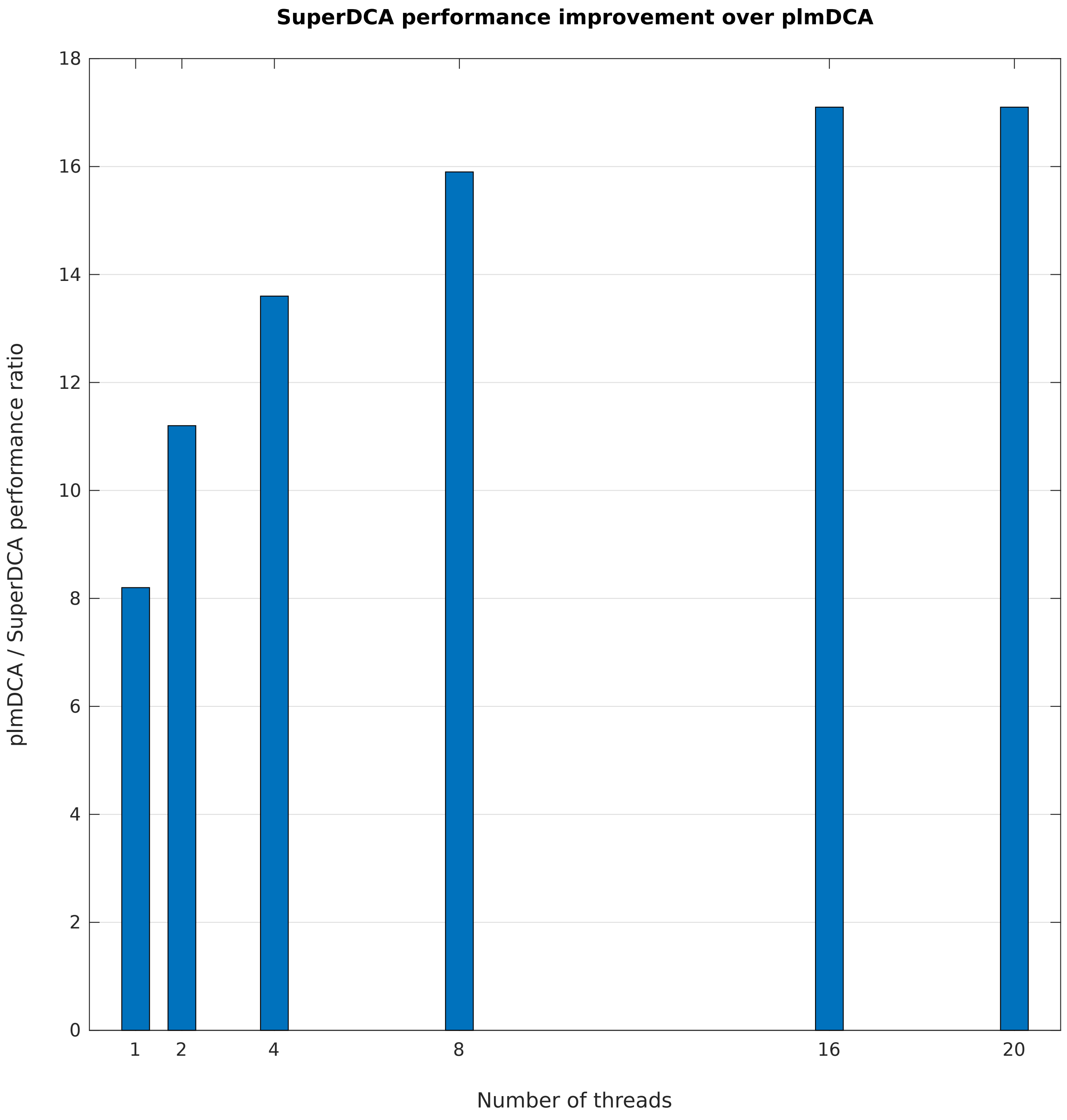

Supplementary Figure 9. SuperDCA runtime improvement over plmDCA.

The single- threaded performance of SuperDCA is more than 8-fold that of plmDCA. Due to the greater parallel scalability of SuperDCA the performance delta grows as more compute threads are used, reaching more than 17-fold when run on 20 cores. See caption of Fig. S7 for details of benchmark settings and hardware.
